# Imaging Beads-Retained Prey Assay for Rapid and Quantitative Protein-Protein Interaction

**DOI:** 10.1371/journal.pone.0059727

**Published:** 2013-03-29

**Authors:** Yan Zhou, Wanjin Hong, Lei Lu

**Affiliations:** 1 School of Biological Sciences, Nanyang Technological University, Singapore, Singapore; 2 Institute of Molecular and Cell Biology, Singapore, Singapore; University of Illinois, Urbana-Champaign, United States of America

## Abstract

Conventional Western blot based pull-down methods involve lengthy and laborious work and the results are generally not quantitative. Here, we report the imaging beads-retained prey (IBRP) assay that is rapid and quantitative in studying protein-protein interactions. In this assay, the bait is immobilized onto beads and the prey is fused with a fluorescence protein. The assay takes advantage of the fluorescence of prey and directly quantifies the amount of prey binding to the immobilized bait under a microscope. We validated the assay using previously well studied interactions and found that the amount of prey retained on beads could have a relative linear relationship to both the inputs of bait and prey. IBRP assay provides a universal, fast, quantitative and economical method to study protein interactions and it could be developed to a medium- or high-throughput compatible method. With the availability of fluorescence tagged whole genome ORFs in several organisms, we predict IBRP assay should have wide applications.

## Introduction

Western blot based pull-down assays are widely employed to study protein-protein interaction. A conventional resin mediated pull-down assay typically involves two steps. In the binding step, the resin immobilized bait is incubated with prey in solution or cell lysate. The beads are subsequently washed to remove nonspecifically bound proteins. In the detection step, the bound prey is eluted and subjected to Western blot detection, which consists of polyacrylamide gel electrophoresis separation, immunoblotting, chemiluminescence and film exposure. The conventional assays have several drawbacks. First, large amount of bait and prey proteins are required due to the low overall detection sensitivity. Second, the procedure is time consuming and generally involves 2 days of work. Third, it is difficult to achieve quantitative analysis since the detection step involves many non-linear processes. For example, it has been reported that the chemiluminescence signal and the quantity of substrate follows hyperbolic rather than linear relationship [1]. Furthermore, the film that records the chemiluminescence usually has a limited linear range, although a commercialized CCD camera box designed for this task could solve this issue. Fourth, the result is not quantitatively repeatable since it is impossible to control parameters in the detection step, such as the amount of protein transferred, the degree of immuno-labeling, chemiluminescence intensity and film exposure time etc. Fifth, the assay cannot be scaled up for medium and high-throughput screening. Furthermore, expensive reagents and consumables are spent in the assay, including antibodies, chemiluminescence substrate and X-ray films etc.

Recently, image-based methods have been proposed to solve the problems associated with the conventional pull-down assay. The luminescence-based mammalian interactome mapping (LUMIER) assay tests two transiently co-expressed proteins, in which the bait is fused to luciferase while the prey is fused to an immunoprecipitation tag [2]. After immunoprecipitation of the prey, the interaction is quantified as the chemiluminescence contributed by the luciferase fused bait. In single bead affinity detection (SINBAD) assay, the pull-down assay is conducted in single beads and multiple preys are detected by antibodies conjugated with quantum dots, which are imaged under fluorescence microscope [3]. The fluorescence of GFP fused prey (GFP-prey) was utilized in “bead halo” assay to qualitatively detect equilibrium interaction to bait immobilized on beads [4]. However, the bead halo assay is unable to study protein interactions quantitatively. Inspired by this assay, we found that, a rapid and quantitative interaction assay could be achieved for GFP-preys if the detection stage is conducted by fluorescence microscopy followed by proper image analysis. This method is referred to as imaging beads-retained prey (IBRP) assay and it addresses and at least partially solves the above mentioned problems associated with the conventional pull-down approach.

## Results

**Figure 1:**
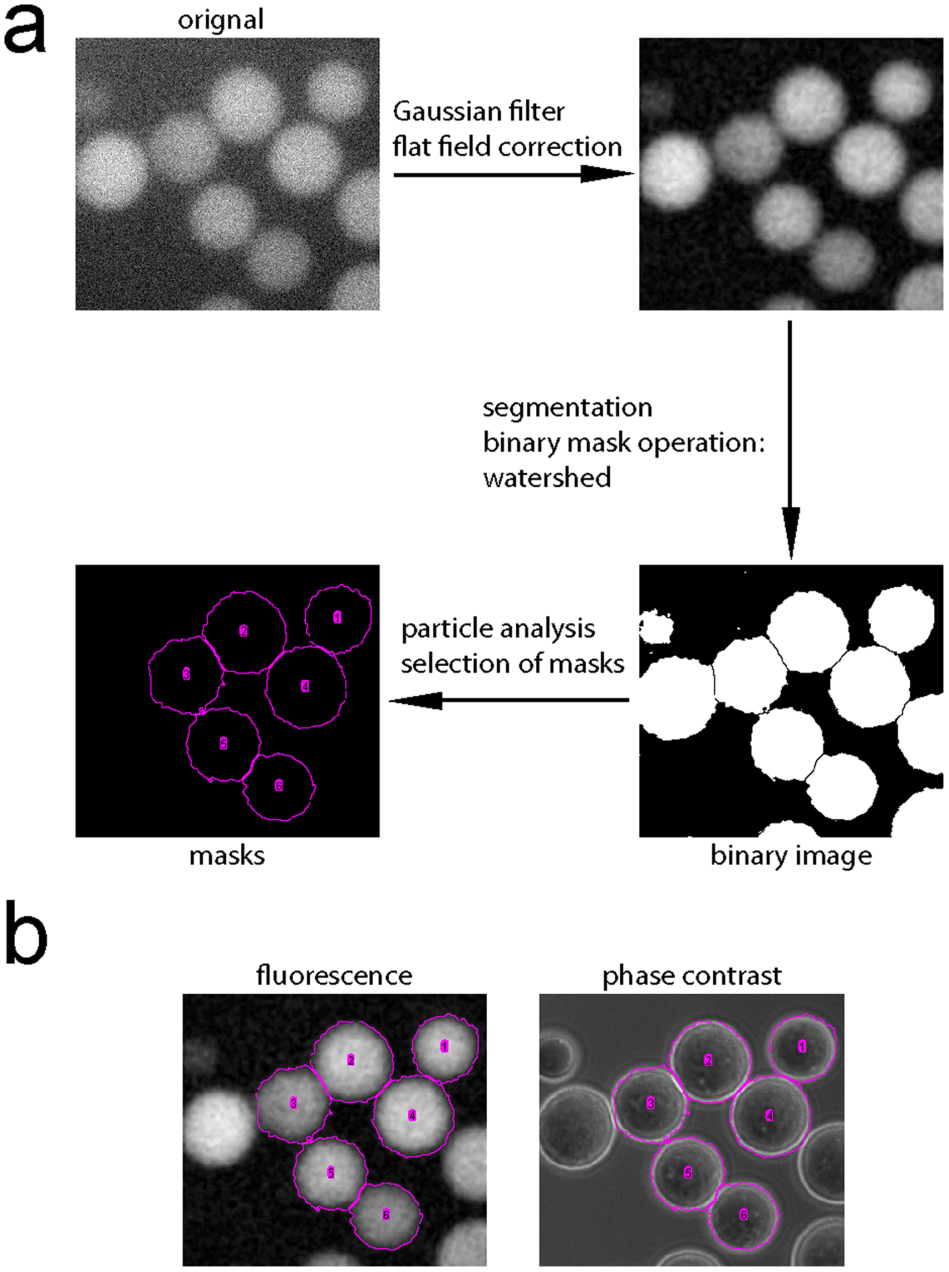
Generating masks for IBRP assay. (a) A method to mask individual beads using ImageJ. The masks are shown as magenta circles labeled with numbers. (b) The resulted masks are overlaid onto fluorescence and phase contrast images of the same beads. The masks are found to match the physical contour of the corresponding beads.

Our IBRP assay requires the usage of GFP (or other fluorescence protein) fused prey, which could be widely available in scientific community or easily created. The binding step of the assay is essentially the same as the conventional GST (Glutathione S-transferase) pull-down assay. The recombinant GST-fused bait protein (GST-bait) is immobilized onto Glutathione agarose beads as normal. Mammalian cells (or any prokaryotic or eukaryotic cells) transiently or stably expressing GFP-prey are lysed and the cell lysate containing GFP-prey is incubated with the GST-bait beads at 4°C. Very small amount of beads, such as <1–2 µl, and a short incubation time, such as 30 min, could be used. After incubation, the beads are extensively washed and, during detection step, an aliquot of beads is loaded onto a microwell on a glass slide and imaged under an inverted microscope. The loading or input of GST-bait is semi-quantified by Coomassie staining and expressed as the amount of protein per volume of beads (µg/µl or µM) (input of GST-bait), while the input of GFP-prey is quantified by measuring the fluorescence intensity of the lysate. Two sizes of Glutathione agarose beads were tried–the small one with a mean diameter of ∼30 µm and the large one ∼90 µm. While each of the small beads have a homogenous distribution of GFP signal, each of the large ones was observed to have a gradually decreased intensity from peripheral to center as previously reported [4]. The difference could be due to the limited diffusion of the GFP-prey to the center of the large beads. Our IBRP assay is not affected by the size of beads. The free image analysis software–ImageJ is utilized to quantify the intensity of GFP-prey on beads. Figure 1a illustrates steps to generate masks for intensity analysis. Briefly, the fluorescent image is subjected to Gaussian filtering to remove noise and flat field correction to alleviate uneven background associated with air objective lens. After segmentation and watershed, the resulted masks are further filtered by criteria such as size, shape (circularity) and positions to exclude aggregated, broken and edge localized beads. The masks generated are in a good agreement with phase contrast image of beads (Figure 1b). The method yields satisfactory results even for images with low signal-to-noise ratios (the image in Figure 1a has a signal-to-noise ratio of ∼ 4). In the worst scenario when the beads have very weak GFP fluorescence, masks could be manually drawn using ImageJ circle tool by tracing phase contrast image. 50–200 beads could be masked from images and the mean intensity of each bead could be measured and averaged. The mean intensity per bead reflects the strength of interaction between bait and prey in IBRP assay. These mask generation steps could be automatically processed by compiling a macro in ImageJ. Images of beads could be acquired at different exposure times to maximize the dynamic range of a CCD camera. Furthermore, assays could have different inputs (loadings) of bait and prey. To compare different assay results and normalize exposure time, input of GFP-prey and input of GST-bait, we introduce IBRP affinity, which is calculated as the follow:
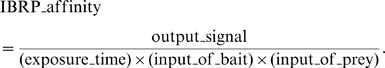



In IBRP assay, the strength of binding between bait and prey is expressed as IBRP affinity. The normalization of exposure time is based on the linear relationship between photonic input and electronic output of scientific CCD camera. The basis for the normalization of bait and prey is discussed later. IBRP affinity is a relative value and therefore in cases when the same bait or prey is used in an assay, the value of that input is assigned as 1. All data presented in this manuscript are normalized by exposure time.

**Figure 2:**
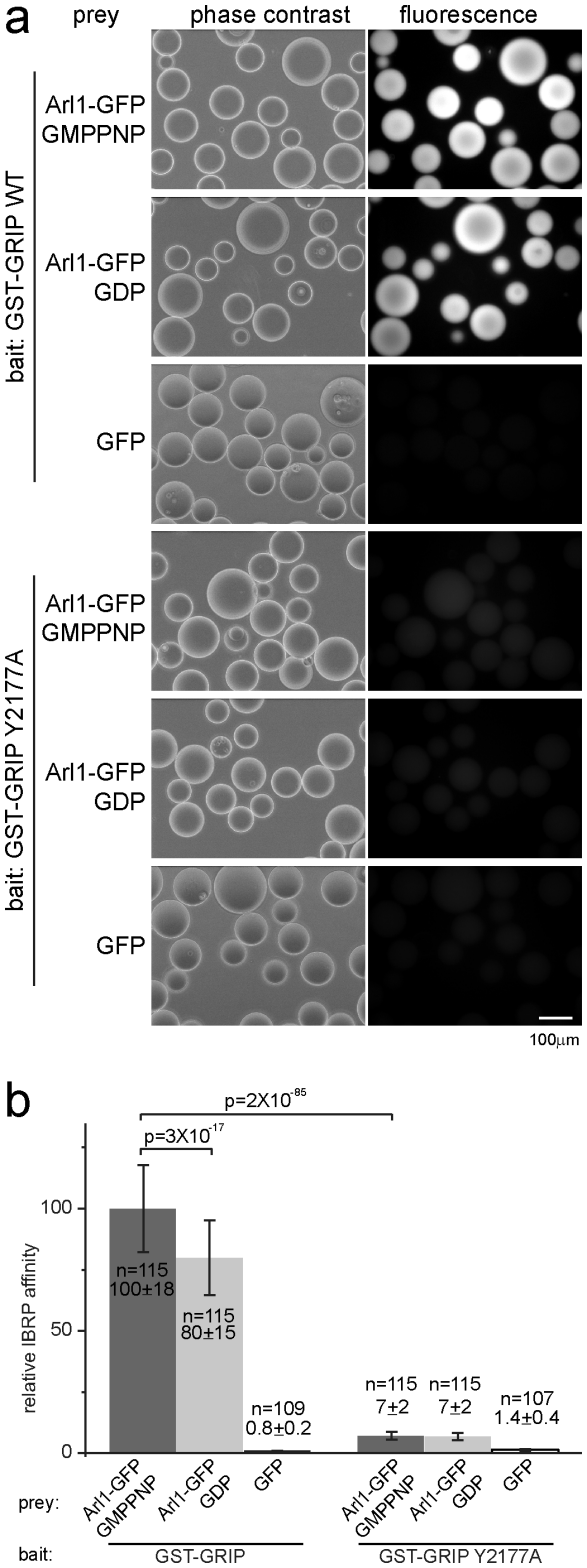
Studying the interaction of Arl1/GRIP by using GST-GRIP as bait and Arl1-GFP as prey in IBRP assay. (a) GST-GRIP or Y2177A mutant was immobilized onto beads at 16 µg/µl. The beads were incubated with the cell lysate containing Arl1-GFP (in the presence of 100 µM GMPPNP or GDP) or GFP (as a negative control) and imaged under phase contrast (left column) or fluorescence (right column) setting. In the right column, the fluorescence images were linearly scaled for a fair visual comparison of intensity. Scale bar, 100 µm. (b) Relative IBRP affinity of each interaction. Error bars represent standard deviations. n indicates the number of beads quantified. p indicates the p value of selected pair calculated by t-test.

As proof of principle, we analyzed a well characterized interaction between GRIP domain of Golgin245 (hereafter referred to as GRIP domain) and Arl1, an ARF family small GTPase. A small GTPase has two guanine nucleotide binding states in cells–GTP (guanosine 5′-triphosphate) bound or active state and GDP (guanosine 5′-diphosphate) bound or inactive state. Previously, we studied the interaction between Arl1 and GRIP domain extensively and solved the crystal structure of Arl1-GTP/GRIP [5], [6]. Our data indicated that the GRIP domain is sufficient and necessary for interacting with Arl1-GTP but not Arl1-GDP [5], [6]. Among various amino acids of GRIP domain, a tyrosine at 2177 was shown to be essential for this interaction [5], [6]. GST-GRIP wild type (GST-GRIP) or Y2177A mutant immobilized on beads was incubated with lysates from cells expressing either GFP (as a negative control) or Arl1-GFP in the presence of either 100 µM GDP or GMPPNP (guanosine 5′-[β,γ-imido]triphosphate; a non-hydrolyzable analog of GTP). Figure 2a shows the fluorescence and corresponding phase contrast images of the beads in IBRP assay. In GST-GRIP panel, while beads incubated with GFP appeared dark, bright fluorescent beads were observed upon incubation with Arl1-GFP in the presence of GMPPNP or GDP. Quantification of the fluorescence intensity of beads indicated that GST-GRIP wild type interacts with more Arl1-GFP in the presence of GMPPNP than GDP (p = 3×10^−17^), as expected by the GTP dependent nature of this interaction (Figure 2b). Even in the prevalent presence of GDP, a significant amount of the GTP bound Arl1-GFP could remain unexchanged to GDP and therefore contribute to the fluorescence in GDP panel. Tyrosine to alanine mutation at position 2177 reduced the Arl1/GRIP interaction by more than 14 folds, quantitatively demonstrated our previous yeast-two-hybrid assays [5], [6]. Collectively, the results of our IBRP assay are consistent with our previous studies and therefore validate this method.

**Figure 3:**
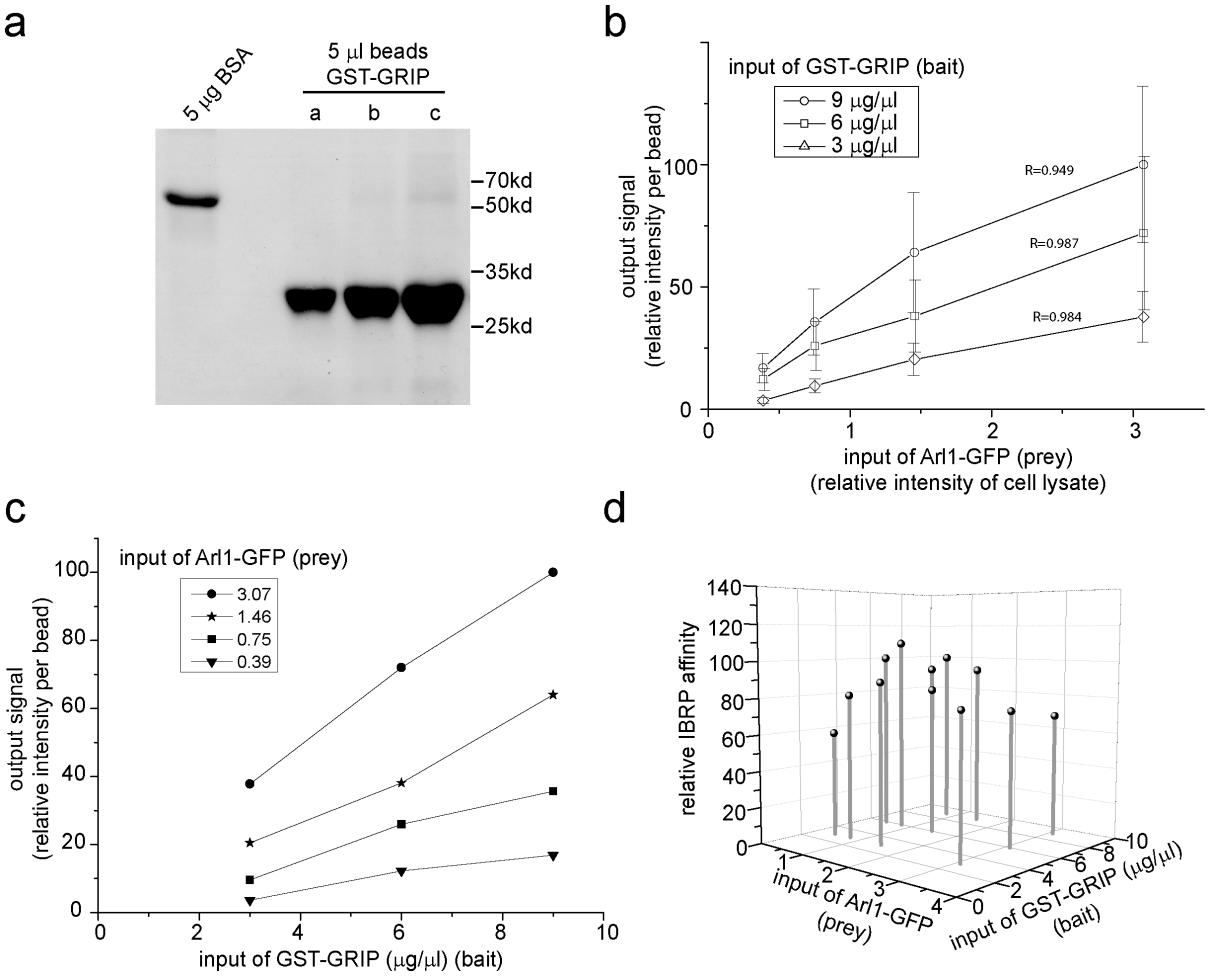
Characterizing the input and output relationship of IBRP assay using Arl1/GRIP interaction. (a) Quantification of the input of GST-GRIP used in this study by Coomassie staining. GST-GRIP bead slurry in preparation a, b and c was semi-quantified as 3, 6 and 9 µg/µl, respectively. BSA, bovine serum albumin. (b-d) The assay was conducted using a combination of various inputs of GST-GRIP (bait) and Arl1-GFP (prey). The number of beads quantified (n) for each data point is in between 95 and 141. The same set of data are plotted in three different formats to illustrate the input and output relationship. (b) The linear relationship between the output signal (relative intensity per bead) and the input of Arl1-GFP (relative intensity of the cell lysate). R indicates the R-square values for linear fitting. Error bars represent standard deviations. (c) The linear relationship between the output signal (relative intensity per bead) and the input of GST-GRIP (µg/µl). (d) The relative IBRP affinity, input of Arl1-GFP and input of GST-GRIP are plotted in a 3D graph. The mean±standard deviation of the relative IBRP affinity is 90±10 (n = 12).

We further quantitatively characterized our IBRP assay in term of the relationship between input and output using the Arl1/GRIP interaction (Figure 3). Here, no exogenous guanine nucleotide was added to the system and we tested the interaction between the immobilized GST-GRIP (bait) and GTP form of Arl1-GFP (prey) present in the cell lysate. Three batches of beads (a, b and c) with different densities of GST-GRIP were prepared and semi-quantified by Coomassie staining (Figure 3a). Cell lysates with different input of Arl1-GFP were obtained by diluting Arl1-GFP lysate using mock transfected one. Under various combinations of inputs of prey and bait, the outputs of the pull-down assays are plotted in Figure 3b–d. The output signal (intensity per bead or the amount of Arl1-GFP retained per bead) shows linear relationship to at least ∼6 folds range of the input of Arl1-GFP (Figure 3b). The output signal also seems linearly proportional to the input of the GST-GRIP in at least ∼3 folds range, despite the rough estimation of GST-GRIP (Figure 3c). Such linear output and input relationship has been repeated by at least two other independent experiments. Figure 3d shows the calculated IBRP affinity. Within ∼3 and ∼6 folds ranges of two inputs, the standard deviation is 17% of the mean relative IBRP affinity. Our finding therefore serves as the basis for the usage of IBRP affinity to semi-quantitatively compare the interaction strength between bait and prey.

**Figure 4:**
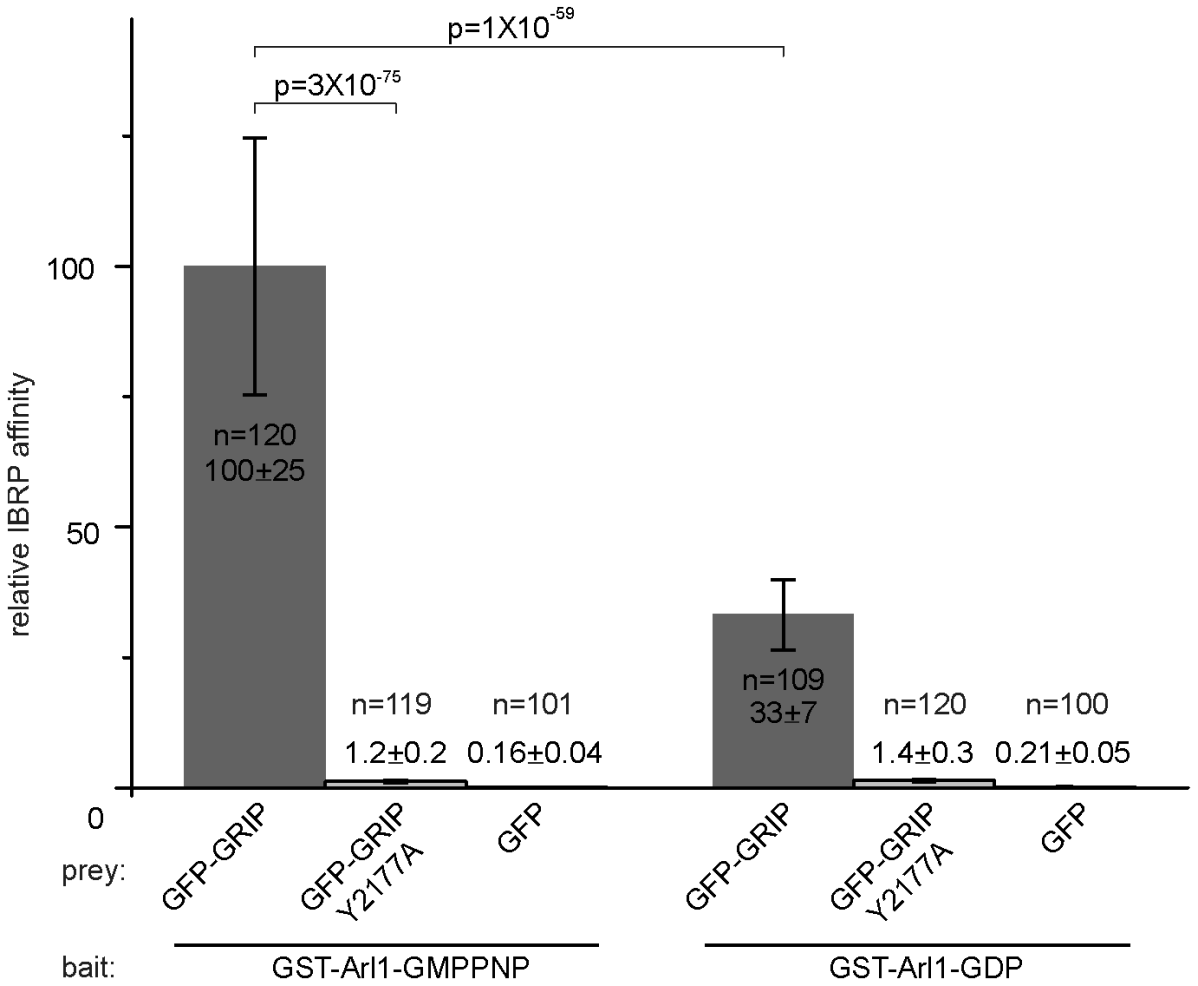
Studying the interaction of Arl1/GRIP by using GST-Arl1 as bait and GFP-GRIP as prey in IBRP assay. GST-Arl1 immobilized on beads (13 µg/µl) was loaded with either GMPPNP or GDP. The beads were incubated with cell lysate containing the following GFP-prey: GFP-GRIP, GFP-GRIP Y2177A or GFP (as a negative control). Relative IBRP affinities were culaculated and plotted. Error bars represent standard deviations. n indicates the number of beads quantified. p indicates the p value of selected pair calculated by t-test.

To further test the IBRP assay, we reversed the bait and prey in Arl1/GRIP interaction. GST-Arl1 immobilized on beads was subjected to *in vitro* guanine nucleotide exchange to load GDP or GMPPNP. The exchanged GST-Arl1 was subsequently incubated with lysate containing GFP (as a negative control), GFP-GRIP or GFP-GRIP (Y2177A). After quantification, the relative IBRP affinity of Arl1/GRIP interaction in the presence of GMPPNP is 3 folds that of GDP (Figure 4). When the critical Y at 2177 of Golgin245 GRIP domain is mutated to A, the relative IBRP affinity between GRIP and Arl1-GMPPNP is reduced ∼80 folds of the wild type GRIP domain. These reverse IBRP data are consistent with results from Figure 2 and our previous studies [5], [6].

**Figure 5:**
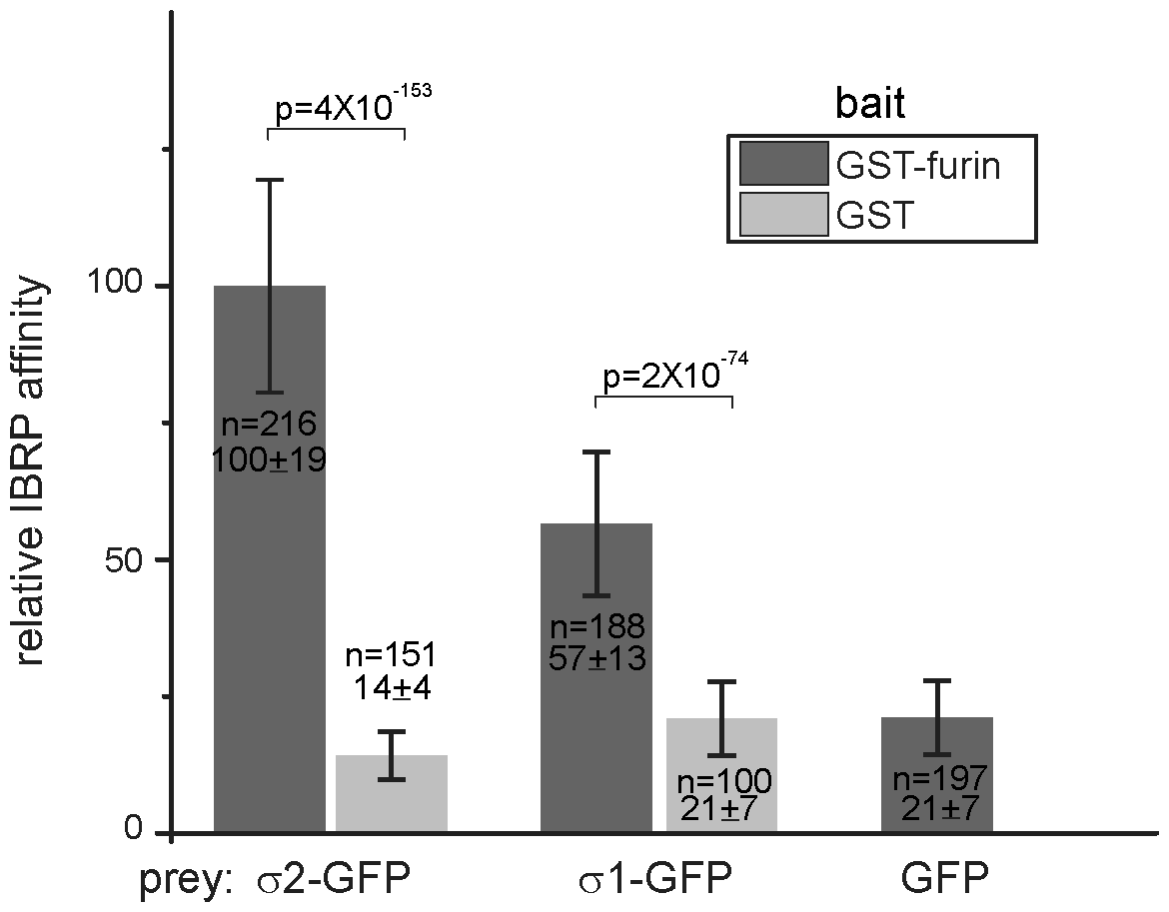
Studying the interaction between furin cytosolic domain and clathrin adaptor proteins AP1 and 2 using IBRP assay. The bead immobilized GST-furin (2 µg/µl) or GST (3 µg/µl, as a negative control) were served as baits to pull down cell lysate containing σ2-GFP, σ1-GFP and GFP (as a negative control). The relative IBRP affinities were calculated and plotted. GST-furin, but not GST, selectively binds σ2-GFP and σ1-GFP. Error bars represent standard deviations. n indicates the number of beads quantified. p indicates the p value of selected pair calculated by t-test.

Our IBRP assay is also applicable to other protein interaction systems. We tested the binding of furin cytosolic domain with adaptor protein (AP) 1 and 2, which are generally believed to link clathrin coats to the cytosolic domains of membrane cargos [7], [8]. The endoprotease Furin is a type I transmembrane protein, whose sub-cellular trafficking is mainly determined by the interaction of sorting motifs in its cytosolic domain with adaptors, such as AP1 and 2 [9]. Compared to other protein-protein interactions, it is known that sorting motifs bind adaptor proteins at low affinity [10]. We cloned the C-terminal cytosolic domain of mouse furin (58 aa) and fused it to the C-terminus of GST. Both AP1 and 2 are labeled by expressing their smallest subunits σ1 and 2-GFP, respectively. Using IBRP assay, both σ1 and 2-GFP, but not GFP, were specifically retained by GST-furin (Figure 5). Under the same condition, σ1 and 2-GFP retained on the control GST beads were ∼50% and 25% of furin, respectively. Our results are in agreement with previous reports and therefore indicate that our IBRP assay should be applicable to study other protein interaction systems.

## Discussion

In this study, we reported and characterized a novel IBRP assay for studying protein-protein interactions. The binding step of this assay is essentially conventional pull-down experiment in a smaller scale, while the detection step is based on fluorescence imaging and analysis. A simple and linear relationship between bait/prey complex and the output signal could be maintained in the detection step of IBRP assay. This could be due to the following two facts. First, the bait/prey complex is directly visualized on beads *vs* indirectly detected by Western blot followed by chemiluminescence. Second, the final measurement is conducted by CCD camera, the dynamic linear range of which is 1–2 orders of magnitude more than that of X-ray film. The IBRP assay features the following advantages comparing to the conventional Western blot based pull-downs. 1) It is easy and rapid to perform as the whole experiment could be done in half a working day. 2) It requires a small amount of bait and prey–∼50 beads with immobilized bait and a fraction of the 35 mm Petri-dish cells expressing GFP-prey could be sufficient. 3) It is highly quantitative and the IBRP affinity could be used to compare assays conducted in parallel. The IBRP affinity should be repeatable if each step is carefully controlled. 4) It does not require equipments, such as polyacrylamide gel running and transfer apparatus, chemiluminescence detection CCD box, film processor and densitometer. A low end inverted epifluorescence microscope with a cooled CCD camera is sufficient. The analysis software, such as ImageJ, is freely available with strong support from scientific community. It is economical as it avoids the use of consumables, such as protein transfer membrane, antibodies, chemiluminescence substrate and X-ray film etc, and saves the expensive maintenance cost of the film developer. 5) It is suitable for other fluorescence proteins and tags, such as mCherry, Halo and SNAP tags. The bait could also be immobilized onto beads by other affinity tags, such as His or MBP (maltose-binding protein), or by covalently cross-linking to solid support. It is possible to study the multiplexed protein-protein interactions in a single IBRP assay when various fluorescence color-coded preys are incubated with various baits immobilized onto size-coded beads. 6) It could be adapted to automatic medium to high throughput screening.

There are mainly three beads-based pull-down assays in the literature. Although each of the three methods has its merits, IBRP assay provides a good alternative to them. In camparison to “bead halo” assay [4], which inspired our assay, IBRP assay is able to quantitatively detect protein interactions. Unlike SINBAD assay [3], IBRP assay does not require antibodies of preys which could be difficult to obtain. Our assay also directly detects the bound prey by fluorescence instead of indirect chemiluminescence utilized in LUMIER assay [2]. As an *in vitro* pull-down assay, IBRP assay requires recombinantly purified bait and fluorescently labeled/fused prey. For a bait protein with transmembrane domain(s), a soluble region must be determined as the bait. Similar to “bead halo” and yeast-two-hybrid assay, the fusion of a large protein tag to a prey could potentially interfere with functions of the prey in IBRP assay. In some cases, the fusion of a tag at one particular end (N- or C- terminus) could abolish the cellular function of a prey. For example, when GFP is fused to the N-terminus of Arl1, the resulting GFP-Arl1 is non-functional in cells [11]. Therefore, both N- and C-terminus tagged prey libraries should be tried for characterizing or screening unknown interactions. IBRP assay should work for preys with transmembrane domains under appropriate solubilization protocol as in the conventional pull-down assays. With the wide usage of fluorescent techniques, more and more proteins are genetically fused to fluorescence proteins or tags. For example, the genes of some organisms, such as yeast (Invitrogen) and fly [12], have been systematically tagged with GFP. Furthermore, fluorescence tagged expression-ready clones of whole genome ORFs, including human, mouse and zebra fish, are currently commercially available (http://www.genecopoeia.com/tech/omicslink/). We believe that the IBRP assay could become a general method for studying protein-protein interactions.

## Materials and Methods

### Cloning

Wild type rat Arl1 was cloned to pEGFP-N1 (Clontech) using standard PCR technique. The template for the PCR was Arl1 wild type cloned in pSTAR vector [11]. GST-Arl1 has been previously described [6]. Mouse furin cytosolic domain (58 amino acids including from QLRSG to DQSAL) was amplified from mouse Testis Marathon Ready cDNA Library (Clontech) using Taq DNA polymerase (Applied Biosystems) and cloned into EcoRI/BamHI sites of pGEB [6], a modified pGEX-KG vector (GE Healthcare). The following plasmid constructs were described previously [5]: Golgin245 GRIP domain wild type and Y2177A mutant in pEGFP-C2 (Clontech) and Golgin245 GRIP domain in pGEX-6P1 (GE healthcare) (referred to as GST-GRIP). Golgin245 GRIP Y2177A in pGEB (GST-GRIP Y2177A) was cloned by digesting Golgin245 GRIP Y2177A in pEGFP-C2 by EcoRI/BamHI and cloned into pGEB using the same sites. σ1 and 2 in pEGFP-N1 are generous gifts from Tomas Kirchhausen (Harvard Medical School, Boston).

### Preparation of GST-bait Immobilized on Glutathione Beads

Plasmid constructs (pGEB or pGEX-6P1) for GST-Arl1, GST-GRIP (wild type or Y2177A) and GST-furin were transformed into BL21 *E coli* cells. After induction by Isopropyl β-D-1-thiogalactopyranoside, bacterial pellet was lyzed by sonication in bacteria lysis buffer (50 mM Tris pH 8.0, 0.1% Triton-X 100, 5 mM DTT, 1 mg/ml lysozyme) supplemented with phenylmethanesulfonyl fluoride and complete protease inhibitor (Roche). After high speed centrifugation, the supernatant was incubated with Glutathione Sepherose 4B beads (GE Healthcare; Catalog number 17-5279-01 and 17-0756-05 for small and large beads, respectively) at 4°C overnight. The bead slurry was washed three times with 50 mM Tris pH 8.0, 0.1% Triton-X 100 and the bound GST fusion proteins were semi-quantified by SDS-PAGE using bovine serum albumin standard loaded in parallel. Beads were stored at 4°C until use.

To exchange the guanine nucleotide of the bead immobilized GST-Arl1, GST-Arl1 bead slurry was washed with buffer (20 mM Hepes pH 7.3, 100 mM NaCl) twice and incubated with the exchange buffer (20 mM Hepes pH7.3, 100 mM NaCl, 5 mM EDTA, 1 mM DTT) supplemented with 1 mM GMPPNP (guanosine 5′-[β,γ-imido]triphosphate) or GDP (gunosine 5′-diphosphate) (Sigma) overnight at 4°C. The exchange reaction was stopped by adding Mg^2+^ to a final concentration of 10 mM.

### Preparation of the Cell Lysate Containing GFP-prey

HEK293 cells were plated onto 6-well plates or 35 mm Petri-dishes and grown in DMEM supplemented with 10% fetal bovine serum. Cells were transfected by GFP constructs using Lipofectamine 2000 (Invitrogen) according to standard protocols. One day after transfection, cells were lyzed by 150 µl lysis buffer (1% TritonX-100, 20 mM Hepes pH7.3, 100 mM NaCl, 1 mM DTT and complete protease inhibitor). The crude lysate was subsequently cleared by centrifugation at maximal speed in a table top centrifuge at 4°C and the supernatant was saved.

### IBRP Assay

Before the binding reaction, beads were blocked by incubating with lysis buffer containing 4 µg/µl bovine serum albumin. 2 µl blocked bead slurry was subsequently incubated with 50 µl cell lysate for 30 min at 4°C. The beads were washed five times by low speed centrifugation with washing buffer (20 mM Hepes pH 7.3, 0.1% TritonX-100 and 300 mM NaCl), re-suspended in phosphate buffered saline and loaded into 64-well glass slide formed by adhering a piece of multiwell silicone gasket (Grace Bio-Labs) onto a piece of glass slide. The microscope employed in imaging had an inverted design (Axiovert 200 M, Zeiss) with the following configurations–a phase contrast 20× objective (Plan-neofluar and N.A. 0.50), GFP fluorescence filters, cooled CCD camera (Coolsnap HQ, Photometrics) with a 0.65× adaptor lens and a Xenon lamp (X-cite, Lumen Dynamics). The microscope system was measured to have a total magnification of 13× and a field of view of 0.7×0.5 mm. The beads, which settled onto the bottom of the well, were imaged under the setting of GFP fluorescence and phase contrast. To determine the input of GFP-prey, 8 µl of cleared cell lysate was loaded into a well of 64-well glass slide. The bottom of the well was subsequently imaged and the mean GFP fluorescence intensity was calculated.

### Image Analysis

Image analysis was processed in ImageJ (http://imagej.nih.gov/ij/). To generate masks of individual beads as shown in Figure 1, the GFP fluorescence images were first Gaussian filtered (radius = 1 pixel). The uneven field was corrected by rolling ball background subtraction (Process→subtract background→rolling ball) using a radius of 400 pixels and the resulted even background image was duplicated as image A and B. Image A was subsequently segmented and the resulting binary image was further subjected to watershed treatment (Process→Binary→Watershed) and particle analysis (Size (pixeĺ2): 200-infinity; Circularity: 0.60–1.00; “Exclude on edges”: selected; “Add to manager”: selected). After visual inspection, these masks were used to measure the mean intensities of individual beads using image B. The mean intensity of individual bead was corrected by background and normalized by exposure time. If the intensity was weak it was necessary to further correct it by subtracting the auto-fluorescence of blank Glutathione beads. The input of the GFP-prey was similarly normalized by exposure time and corrected by subtracting the auto-fluorescence of mock transfected cell lysate. In each set of data, the IBRP affinity was calculated as described in results. Relative IBRP affinities were used for plotting by normalizing data to make the max value 100. The statistic analysis was conducted in Excel or OriginPro 8.5 (Origin Lab). Student t-test was performed in Excel by assuming two-tailed distribution and unequal variance. The signal to noise ratio was calculated as the ratio of the mean intensity of beads to the standard deviation of the background.
